# Expression of genes involved in the T cell signalling pathway in circulating immune cells of cattle 24 months following oral challenge with Bovine Amyloidotic Spongiform Encephalopathy (BASE)

**DOI:** 10.1186/s12917-015-0412-y

**Published:** 2015-05-09

**Authors:** Andrea Trovato, Simona Panelli, Francesco Strozzi, Caterina Cambulli, Ilaria Barbieri, Nicola Martinelli, Guerino Lombardi, Rossana Capoferri, John L Williams

**Affiliations:** Parco Tecnologico Padano, via Einstein, Lodi, 26900 Italy; Istituto Sperimentale Italiano Lazzaro Spallanzani, Loc. La Quercia, 26027 Rivolta d’Adda, Italy; Istituto Zooprofilattico Sperimentale della Lombardia e dell’Emilia Romagna, via Bianchi 9, 25124 Brescia, Italy; Present address: School of Animal and Veterinary Sciences, University of Adelaide, Roseworthy, SA 5371 Australia

**Keywords:** Bovine Amyloidotic Spongiform Encephalopathy (BASE), Transmissible Spongiform Encephalopathies (TSEs), Immune function, Cattle

## Abstract

**Background:**

Bovine Amyloidotic Spongiform Encephalopathy (BASE) is a variant of classical BSE that affects cows and can be transmitted to primates and mice. BASE is biochemically different from BSE and shares some molecular and histo-pathological features with the MV2 sub-type of human sporadic Creutzfeld Jakob Disease (sCJD).

**Results:**

The present work examined the effects of BASE on gene expression in circulating immune cells. Ontology analysis of genes differentially expressed between cattle orally challenged with brain homogenate from cattle following intracranial inoculation with BASE and control cattle identified three main pathways which were affected. Within the immune function pathway, the most affected genes were related to the T cell receptor-mediated T cell activation pathways. The differential expression of these genes in BASE challenged animals at 10,12 and 24 months following challenge, vs unchallenged controls, was investigated by real time PCR.

**Conclusions:**

The results of this study show that the effects of prion diseases are not limited to the CNS, but involve the immune system and particularly T cell signalling during the early stage following challenge, before the appearance of clinical signs.

## Background

BSE (Bovine Spongiform Encephalopathy) is a fatal neurodegenerative disorder that affects cattle, which was first identified in 1986 in the UK [1]. Following the first description of BSE, the number of cases rapidly increased and reached epidemic proportions in the UK cattle population. A relatively small number of BSE cases were also found in cattle in other countries. In 1990 the appearance of a new variant of Creutzfeldt Jakob disease (vCJD) in humans was linked to food borne transmission of BSE, which caused major concerns for public health [2].

The key event in the pathobiology of BSE and other Transmissible Spongiform Encephalopathies (TSEs) is the conversion of the cellular prion protein (PrP^c^) into an insoluble, protease-resistant isoform, PrP^res^. This aberrant form of the protein eventually accumulates in the central nervous system (CNS) and is associated with the onset of clinical disease [3]. In the initial phases of classical transmissible prion diseases, such as scrapie and TSE infection via the oral route, PrP^res^ propagates in the peripheral lymphoreticular system before transmission to the CNS [4]. Progression of classical TSE disease requires the presence of functionally active immune cells, however, the absence of functional lymphocytes does not impair prion pathogenesis and spread to the CNS [5,6]. The pathobiology of spontaneous and atypical prion diseases, however, is not well understood.

The primary biological function of PrP^c^, a surface glycoprotein encoded by the PRNP gene, is still unclear. Many studies suggest that it may play a role in the regulation of ion channels and neuronal excitation [7]. PrP may also be linked to immune function [6,8-10] as PrP^c^ is expressed by several immune cell subsets, including T and B lymphocytes, CD34^+^ hematopoietic precursors, dendritic cells (DC), natural killer cells (NK), granulocytes and monocytes [11-13]. The highest expression of PrP^c^ is observed in a sub-population of T lymphocytes, the CD4^+^ CD25^+^ FoxP3^+^ T regulatory cells [14]. PrP^c^ is implicated in several immune processes, including T cell activation and differentiation, immune memory, monocyte activation, inflammation, DC differentiation and activation, and apoptosis of antigen presenting cells (APC) [8,9,15]. PRNP knock-out mice display impaired renewal of the CD34+ cell precursor pool, an abnormal inflammatory response and phagocytosis, limited capacity of DC to act as APC and impaired T cell activation in response to Concanavalin A (Con-A), which requires a functional T cell receptor (TCR) pathway [8,16]. Other studies have shown that PrP^c^ interacts with the TCR in the activation of T cells [9,17,18].

All the cases of BSE identified during the major outbreak in the UK were of the same strain type [19]. However, an atypical form of BSE, Bovine Amyloidotic Spongiform Encephalopathy (BASE), was discovered in Italy in 2004 in two old (11 and 15 year old) asymptomatic cows *post mortem* [19]. Other atypical forms of BSE were subsequently reported in France, Germany and Japan [19-22]. The frequency of atypical BSE may be similar to the occurrence of sporadic CJD, which is about 1 per million individuals [23]. BASE can be biochemically differentiated from BSE by the different mobility of PrP fragments on gel electrophoresis. BASE can also be distinguished from BSE histo-pathologically based on differences in the distribution of vacuoles in the brain. BASE shares molecular and histo-pathological features with the MV2 sub-type of human sporadic CJD (sCJD) [19,22]. BASE has been experimentally transmitted to cattle, primates, and mice [24-26].

In an earlier study [27] we identified genes differentially expressed between healthy cattle and cattle orally challenged with BASE at 12 months post challenge by microarray analysis. The present work examines samples from the same experimental oral challenge of cattle with BASE at additional time points post challenge, prior to the onset of disease, to assess the effects of the challenge on the expression of genes related to the T cell receptor pathway in circulating immune cells.

## Methods

### Animal resource and RNA preparation

Eleven Holstein heifers of approximately 4 months of age were orally challenged with 50 g brain homogenate from cows inoculated intracranially with BASE (see reference [27]). Challenged animal were regularly clinically monitored and blood (10 ml in EDTA) was collected at 3 month intervals from all animals from 6 months to 24 months post challenge. Ten, age and sex matched Holstein cattle sourced from two commercial farms were used as controls in the analyses. These control animals were deemed free from any obvious disease by veterinary examination.

Animal experimentation was carried out following internal ethical approval of the Istituto Sprimentale Zooprofilattico of Lombardy and Emilia Romagna, and in compliance with the legislation pertinent at the time that the BASE infection and sample collection was carried out, namely European Directive 86/609 and the Italian regulation dl 116/92. Experimental protocols were designed to respect the principle of the 3Rs and to ensure that any suffering was kept to a minimum. BASE challenged animals were inspected daily by qualified veterinary staff for signs of distress, and culling of challenged animals was carried out for sample collection for the parent study using established humane procedures.

Fresh blood was centrifuged at 250 g for 20 minutes, the buffy coat was transferred to a new tube and contaminating red blood cells were lysed with 10 ml of RBC Lysis Solution (5 Prime). RNA was extracted immediately using TRI-reagent (Sigma-Aldrich) following the instructions of the supplier. RNA obtained was quantified using a NanoDrop spectrophotometer (ThermoScientific) and quality-checked using a Bioanalyser 2100 (Agilent).

### Microarray and pathway analysis at 12 months post-infection

Samples from 5 animals randomly chosen among the 11 challenged animals at 12 months post challenge (MPC), together with samples from five breed, age and sex matched healthy control Holstein cattle which were used in the analysis.

About 1 μg of RNA was amplified and labelled with Cy5-ULS following the manufacturer’s protocols (ampULSe Cat. No. GEA-022; Kreatech biotechnology). The purified aRNA was quantified using a NanoDrop spectrophotometer and 4 μg were fragmented to a uniform size, then hybridized to a custom Bovine 90 K array (see [27] for array details). The hybridized arrays were scanned with a GenePix 4000B microarray scanner (Axon, Toronto, Ca) and images, in TIF format, were exported to the CombiMatrix Microarray Imager Software for hybridization quality verification and spot definition. Data were then extracted, loaded into R using the Limma analysis package and signal intensities were analyzed using the standard procedure of the Bioconductor suite [28]. The list of differentially expressed (DE) genes was generated using the linear modeling analysis in Limma, with an adjusted P-value cut-off equal to 0.01.

A bioinformatics pipeline was created in PERL to connect the gene ID (Ensembl ID, GenBank ID or UniProt ID) with known pathways, using the information available from the Kyoto Encyclopedia of Genes and Genomes (KEGG) database.

### Gene expression along the time course of infection

Confirmation and time-course studies were performed by quantitative reverse transcription PCR (qRT-PCR) using samples from four of the five orally challenged cattle used for the array analysis (insufficient material was available from the 5^th^), and four different negative control Holstein cows obtained from a second BSE negative farm. The kinetics of expression of six selected DE genes (TCR delta chain, TRAT1, CD3E, ZAP70, LAT and LCK) was examined at 10, 12, 24 MPC.

RNA samples were treated with RNase-free DNase (Sigma Aldrich) for 15 minutes at room temperature and then used as a template for first-strand cDNA synthesis using the SuperScript® III First-Strand Synthesis System for RT-PCR (Invitrogen) according to the manufacturer’s instruction. Primers for qRT-PCR analysis were designed using Primer Quest (Integrated DNA Technologies) and are shown in Table 1.

**Table 1:** **Primer used for qPCR**

**Gene**	**Function**	**Sequence**
**TCR delta chain**	*Antigen receptor of T cells*	Forward 5′TCGCTTGTTTGGTGAAGGA
		Reverse 5′CCCAGGTGAGATGGCAATAG
**TRAT1**	*TCR associated membrane adapter 1: TCR-mediated T cell activation cascade*	Forward 5′GTGAACAAACTGCAAGACGC
		Reverse 5′CTGGGCTTTCTTCGCTTCC
**CD3E**	*Marker of thymocytes and peripheral T lymphocytes*	Forward 5′TCTGGGACTCTGCCTCTTATTA
		Reverse 5′CAAACTCTCTAGGGCATGTCAG
**LAT**	*Linker for activation of T cells, transduction of the activation signal downstream CD3*	Forward 5′GGAGTCGGGAATATGTGAATGT
		Reverse 5′CTGGGAATTCTGGGTGTCAG
**ZAP70**	*Associated with CD3Z chain; transduction of the activation signal downstream CD3*	Forward 5′CTCATGGCTGACATCGAACT
		Reverse 5′CCACGTCGATCTGCTTCTT
**LCK**	*lymphocyte-specific protein tyrosine kinase*	Forward 5′GACAGCACCAGAAGCCATTA
		Reverse 5′GCGACCATGAGTGACAATCT
**B2MG**	*Beta-2-microglobulin precursor*	Forward 5′CAGCGTCCTCCAAAGATTCA
		Reverse 5′ACCCATACACATAGCAGTTCAG
**ACTB**	*Beta-Actin*	Forward 5′AGTCCTTTGCCTTCCCAAAA
		Reverse 5′AAGCGATCACCTCCCCTGT

B2MG and ACTB were used as internal controls.

Real Time PCR was performed on a Applied Biosystems (ABI) PRISM 7900HT in 10 μl reactions containing 5 μl of Power SYBR®Green (Applied Biosystems), 0.2 μl of each primer at 10 μM and 3.6 μl of water. The thermal program was, 95°C for 10 min, then 40 cycles of amplification including two steps: 15 s at 95°C, 30s at 58°C, 30s at 60°C. Each reaction was performed in triplicate.

## Results and discussion

### Identification of differentially regulated genes, qRT-PCR validation and pathway analysis

The analysis of gene expression in white blood cells from 5 cattle 12 months after oral challenge with BASE, identified 140 genes differentially expressed (DE) between BASE challenged and control animals, with a log_2_ fold change of 1.5 or greater and a p value < 0.01. The majority of DE genes (91) were up-regulated in the BASE animals compared with controls. Gene ontology analysis using the KEGG Database identified 34 genes that fell in 3 pathways each with several genes showing affected expression (Tables 2, 3 and 4). The pathway with the largest number of affected genes was related to immune function (21) followed by signal transduction and cell growth (8) then genes coding for metabolic proteins (5). The microarray data set supporting the results of this article is available in the NCBI GEO data repository with accession number GSE67576, [see http://www.ncbi.nlm.nih.gov/geo/query/acc.cgi?acc=GSE67576].

**Table 2:** **Differentially expressed Immune related genes**

**Sequence ID**	**Gene name and function**	**Log fold change**	**P value**
ENSBTAG00000005892	ZAP70, Zeta-chain (TCR) associated protein kinase 70 kDa	1.81	0.001924
ENSBTAG00000002259	TCRβ, T Cell Receptor Beta Chain	2.66	0.000014
ENSBTAG00000000431	TCRδ, T Cell Receptor Delta Chain	3.18	0.000025
ENSBTAG00000000183	TRAT1, T cell receptor associated transmembrane adaptor 1	2.26	0.000003
ENSBTAG00000011359	CD7, T Cell Antigen CD7	1.75	0.000607
ENSBTAG00000001002	TCF7, T Cell Specific Transcription factor 7	3	0.000025
ENSBTAG00000015710	CD3E, T Cell surface antigen CD3 epsilon chain	3.21	0.000010
ENSBTAG00000021249	LAT, Linker for activation of T cell	2.79	0.000010
ENSBTAG00000012695	LCK, lymphocyte-specific protein tyrosine kinase	1.54	0.000532
ENSBTAG00000006453	CD3g, , T Cell surface antigen CD3 gamma chain	2.11	0.000058
ENSBTAG00000030425	ID3, inhibitor of DNA binding 3, dominant negative helix-loop-helix protein	1.97	0.000710
ENSBTAG00000005990	S1PR1, sphingosine-1-phosphate receptor 1	1.5	0.001691
ENSBTAG00000020319	ALOX5, arachidonate 5-lipoxygenase	−1.85	0.002847
ENSBTAG00000011990	ALOX12, arachidonate 15-lipoxygenase	−2.15	0.000439
ENSBTAG00000001321	Il1β, Interleukin 1 beta	−1.91	0.000644
ENSBTAG00000019428	CCR1, chemokine (C-C motif) receptor 1	−1.41	0.001837
ENSBTAG00000038042	IL8βR, chemokine (C-X-C motif) receptor 2 (CXCR2)	−1.96	0.004877
ENSBTAG00000003305	NCF1, neutrophilcytosolicfactor 1	−2.04	0.000868
ENSBTAG00000037735	C5L2, G protein-coupled receptor 77 (GPR77)	−1.76	0.000958
ENSBTAG00000027051	PTAFR, platelet-activating factor receptor	−1.41	0.000081
ENSBTAG00000004322	FOS, murine osteosarcoma viral oncogene homolog	−1.57	0.000152

**Table 3:** **Differentially expressed Metabolic Pathway genes**

**Sequence ID**	**Gene name and function**	**Log fold change**	**P value**
ENSBTAG00000000065	(CRLS1), cardiolipinsynthase 1	1.52	0.0017
ENSBTAG00000031814	SDH, serine dehydratase	−3.55	0.0014
ENSBTAG00000001154	DGAT2, diacylglycerol O-acyltransferase 2	−2.42	0.00044
ENSBTAG00000012855	LPL, lipoproteinlipase	−1.7	0.00045
ENSBTAG00000009733	F16P1, fructose-1,6-bisphosphatase 1 (FBP1)	−1.64	0.00733

**Table 4:** **Other differentially expressed genes**

**Sequence ID**	**Gene name and function**	**Log fold change**	**P value**
ENSBTAG00000013761	STMN1, stathmin 1	1.66	0.00002
ENSBTAG00000008436	CDC25B, Cell division cycle 25B	2.01	0.00003
ENSBTAG00000009663	CSDA, cold shock domain protein A	1.7	0.00015
ENSBTAG00000007336	HCST, hematopoietic cell signal transducer	1.86	0.0003
ENSBTAG00000020350	DUSP2, dualspecificityphosphatase 2	−1.63	0.00264
ENSBTAG00000005947	PLAU, plasminogenactivator, urokinase	−1.61	0.00012
ENSBTAG00000039657	H2A, Histone 2°	−1.5	2.5E-05

### Immune response related genes showing altered expression 12 MPC with BASE

#### TCR signalling cascade

The pathway with most affected genes at 12 MPC was related to immune function with 21 DE genes, of these 11 belonged to the TCR signalling cascade which regulates the activation of T lymphocytes in response to antigen presented by the Major Histocompatibility Complex (MHC). Three genes involved in this pathway with differential expression (TCRβ, TCRδ, and TRAT1) were missing from the KEGG database for cow and were added manually. The pan-T cell marker, CD7, and the transcription factor TCF7/LEF were also added to the TCR pathway by manual annotation. Ten of the DE genes in the TCR cascade were up-regulated, several of which had the highest log-fold change in expression observed at 12 MPC. One gene, the transcription factor Fos, was down regulated, and is downstream of the signalling cascade.

### Kinetics of expression for genes involved TCR signalling in early phases of BASE infection

As TCR signaling is central to immune function and was affected by BASE challenge, the expression of six genes involved in this pathway (TCRδ, CD3E, ZAP70, TRAT1, LAT, LCK) was analyzed over a time course following BASE challenge (10-12-24 MPC) by qPCR. The qPCR analysis confirmed the micro-array data for TCRδ, CD3E, ZAP70 and TRAT1 which were overexpressed in BASE animals vs controls at 12 MPC (respectively p < 0.01; p < 0.01; p < 0.01; p < 0.01). The LAT gene also showed an increase of expression at 12 MPC but the difference compared with controls was not significant.

**Figure 1:**
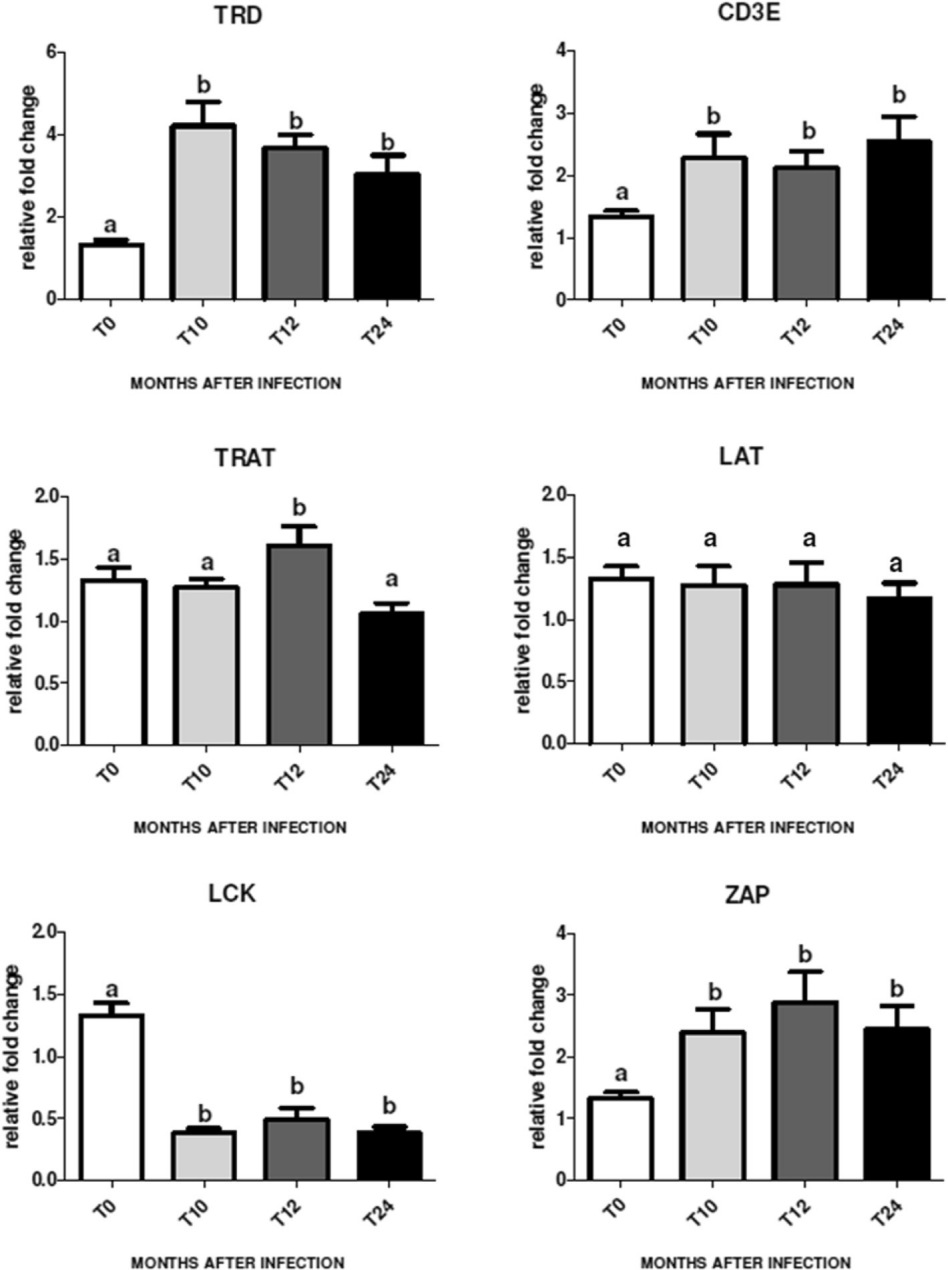
Kinetics of expression for genes involved in the TCR pathway. Results from qPCR analysis of the expression of six genes (TRD, CD3E, TRAT, LAT, LCK, ZAP70) involved in TCR signaling in circulating immune cells, in control animals (T0) and BASE challenged animals at 10-12-24 months post challenge (T10, T12 and T14 respectively). Each column represents the mean ± SEM of at least three separate measurements on 4 individuals. The expression of mRNA normalized to two endogenous reference genes (β-Actin and β-2 Microglobulin), was analyzed by RT-PCR using specific primers as described in Material and Methods. The different *superscripts* indicate significant difference between columns (p < 0.01).

Expression of TCRδ, ZAP70 and CD3E was significantly higher in all 4 infected animals examined by qPCR compared with controls at all the time points post challenge. TRAT1 and LAT showed the same trend, with an increase in expression at 10 MPC and 12 MPC. TRAT 1 had statistically significant increased expression at 12 MPC (p < 0.01), then fell to the same level as control samples by 24 MPC. LCK was found to be up-regulated 1.5 fold in the microarray data at 12 MPC but in the qPCR analysis was down-regulated at 12 MPC (p < 0.01), and also had lower expression than controls at 10 and 24 MPC (see Figure 1).

Interestingly, PrP^c^ has been found to co-precipitate with the TCR [8,18] and with components of the TCR signaling pathway [9], many of which appear among the DE genes identified in the present study (ZAP70, LCK, CD3E, LAT). The physiological function of PrP^C^ is not fully understood, however, it has been implicated in T cell activation after the binding of the antigen [17]. T cell activation increases PrP^c^ concentration on the surface of human lymphocytes [9]. In addition antibodies against PrP^c^ block ConA induced lymphocyte proliferation, which requires a functional TCR complex [16]. How the function of PrP is altered with the change in conformation from PrP^c^ to PrP^res^ is unclear, although there are some suggestions that there is a gain in function and not simply a loss. PrP^res^ is able to stimulate MAP kinase signaling in neuronal cells whereas PrP^C^ is not [29]. In, changes in the level of PrP expression are likely to affect cell function e.g. lack of PrP expression on antigen presenting cells affects T cell activation [30], whereas lack of PrP expression on the T cell itself does not inhibit T cell activation. Nevertheless, the level of PrP is increased following activation. Thus perturbed PrP expression or function, as may occur in the change from PrP^C^ to PrP^res^, is likely to change the dynamics of T cell activation and expression of genes associated with the T cell receptor complex. In the present study, expression of TCRδ, ZAP 70, CD3E, LCK and TRAT was found to change between 10 and 24 month after the BASE challenge. qPCR analysis suggested that LCK was down regulated during early infection (10–24 MPC), which is of interest as this kinase is a key regulator of the TCR pathway. Following the interaction between the TCR and CD3, LCK is recruited to the TCR complex, phosphorylates downstream signaling molecules including ZAP70, and activates a phosphorylation cascade that involves LAT and TRAT [31,32]. This suggests that T cell response is indeed affected following BASE challenge.

#### Genes linked to the inflammatory response

The data presented here also indicate that BASE challenge of cattle is associated with a modified inflammatory response. Expression of ALOX12 and ALOX5, which encode proteins that are key effectors of an inflammatory response were down-regulated at 12 MPC. ALOX12 and ALOX5 have a role in chemotaxis and response in tissue damage [33] and are involved in Arachidonic acid metabolism, which is necessary for Leukotrien production. Leukotriens are key effectors of the inflammatory response [33] and are produced by leukocytes, in particular mast-cells. C5L2 is one of the two receptors for the C5a anaphylotoxin, an extremely potent pro-inflammatory peptide [34], which was also down regulated following BASE challenge. Other pro-inflammatory cytokines or their receptors (IL1β, CCR1, IL8βR and NCF1) were also down-regulated.

Sphingosine 1-phosphate receptor 1 (S1PR1) was up-regulated. S1PR1 is involved in the regulation of inflammatory responses, cell migration and differentiation [35], and the receptor for platelet activating factor (PTARF), a key inflammatory mediator and a pattern recognition receptor involved in the uptake of Gram-positive bacteria [36]. The ID3 gene, which codes for an anti-inflammatory cytokine involved in the TGFβ1 pathway, was also up-regulated. The expression of genes in the TGFβ1 pathway has previously been reported to be up-regulated following prion infection of mice and cattle [37,38]. The results presented here are consistent with previous data, and suggest that prion diseases are associated with an inhibition of inflammation [8]. It is therefore interesting that the expression of IL-8Rβ and NCF1, which are directly linked to chemotaxis of neutrophils, was reduced in BASE challenged animals compared with controls. These results are in agreement with previous studies which proposed that PrP^c^ has a role in the modulation of inflammation and phagocytosis [8], which was also seen in studies of PrP null mice [8].

#### NK-mediated cytotoxicity

HCST, a signal transduction protein involved in NK and T cell activity, especially during anti-viral responses, was up-regulated.

### Metabolic and signal transduction genes of WBC affected by BASE at 12 months post-challenge

In addition to immune function related genes, other pathways were affected by BASE infection, including genes involved in energy metabolism and signal transduction, which are discussed here for completeness.

#### Energy metabolism and storage

Several genes involved in energy metabolism and storage of carbohydrates (F16P1, SDHL) and also genes regulating lipid metabolism and signalling (DGAT2, LIPL), were down regulated in BASE challenged cattle compared with controls at 12 MPC. However, cardiolipin synthase 1 (CRLS1), which is involved in mitochondrial membrane function and is predominantly expressed by tissues with high levels of energy metabolism, was up-regulated. SDHL, which is associated with energy balance, has previously been shown to be affected by TSE diseases. Previous work has shown that proteins related to glucose metabolism have aberrant expression in cerebrospinal fluid of sCJD patients [39]. Patients with sporadic CJD have also been shown to have altered levels of proteins associated with the control of glucose [39] and lipid [37] metabolism. Changes in glucose metabolism are known to trigger apoptotic pathways [39] while changes in lipid metabolism and signalling are one of the early changes apparent in many neurodegenerative diseases, including prion diseases [37]. Therefore this response, while linked to BASE challenge in this study, is not likely to be specific for prion disease.

#### Cell signalling genes

Two genes involved in signal transduction (STMN1, CDC25B), which code for proteins of the MAP kinase pathway (MAPK), were up-regulated, while a negative regulator of this pathway (DUSP2) was down-regulated in challenged animals at 12 MPC. MAPK pathways are essential for cell survival and were up regulated. It has been suggested that MAPK has a role in the protective response to cellular oxidative stress [40]. Previous studies have reported that MAPK proteins interact with PrP^c^ [41]. These genes are also known to play an active role in prion disease pathogenesis in nervous tissues and the medulla, with the sequential activation of the various MAPK associated genes during PrP^res^ deposition [37,40]. MAPK pathway genes have been shown to be up regulated in brain tissues of scrapie infected hamster [40] and mice [37], and in the medulla of cattle following oral challenge with BSE [38]. This is consistent with data which showed that PrP^c^ interacts with MAPK proteins [41].

Genes in other signal transduction pathways also showed changes in expression 12 MPC. CSDA is a member of the highly conserved Cold Shock Domain family of DNA binding proteins which is involved in post-transcriptional control of gene expression [42,43] and was up regulated. The gene coding for histone H2A was down-regulated. Histone 2A is known to co-purify with PrP^res^ extracted from the brains of hamsters infected with experimental scrapie [44].

#### Coagulation cascade

Expression of the gene coding for thrombin receptor (F2R) was up regulated, while the plasminogen activator urokinase (PLAU), was down-regulated. Expression of genes involved in coagulation have also been shown to be affected in the brain of cattle incubating BSE [38].

The BASE challenged animals used for the expression analysis remained healthy up to the 24 month time-point examined here. The parent study which provided the samples in still in progress, and therefore further information on the health status of the animals is not yet available. Data presented here are consistent with that from the earlier study [27], and all animals studied displayed a consistent change in gene expression in comparison with controls, suggesting that they were responding to the BASE challenge. It should also be noted that while challenged animals were housed in a containment facility, controls were taken from two commercial farms. Hence controls and challenged animals experienced different environments, which may have resulted in differences in expression patterns. DE genes between the challenged animals and controls were consistent between the two control groups, which experienced different management regimes. The DE genes and pathways identified between controls and challenged animals are consistent with those reported in other TSE infection models as discussed above. This give some confidence in these data and that the effects of orally administered BASE on gene expression are similar to other TSEs.

## Conclusions

The data presented here on gene expression in circulating immune cells following BASE challenge show that response to BASE has similarities with other prion diseases. PrP^c^ is known to have a role in the immune system, indeed it is expressed on DC and is important for inducing the T cell proliferative response [30]. Moreover, PrP^c^ accumulates in the contact point between T cells and DC, and it may have a role in the assembly of the TCR complex [45]. The disease form of this protein (PrP^res^) has been shown to affect the immune system, e.g. eliciting qualitative differences in the responses of T cells [46]. Moreover, macrophages accumulate PrP^res^, and may be involved in the transfer of the disease to the CNS [9,47]. The data presented here are consistent with the hypothesis that the effects of TSE diseases are not limited to CNS, but involve the immune system, especially during the early stages following challenge, before the appearance of clinical signs. Our data suggest that BASE challenge affects the TCR signalling pathway, which has also been shown in mouse knock-out experiments [17]. BASE therefore, in common with other prion diseases, seems to be associated with general cellular stress and impaired immune function. These data, from experimentally challenged cattle, suggest that orally administered BASE affects gene expression in circulating immune cells even in the absence of overt disease.

## Abbreviations

BASE: Bovine amyloidotic spongiform encephalopathy
BSE: Bovine spongiform encephalopathy
CNS: Central nervous system
DE: Differentially expressed (genes)
MPC: Months post challenge
PCR: Polymerase chain reaction
PrP: Cellular prion protein
sCJD: Sporadic Creutzfeld Jakob disease
TSEs: Transmissible spongiform encephalopathies
TCR: T cell receptor
WBC: White blood cells
